# Analyses of Antioxidant Properties, Mineral Composition, and Fatty Acid Profiles of Soy-Based Beverages Before and After an In Vitro Digestion Process

**DOI:** 10.3390/antiox14040411

**Published:** 2025-03-28

**Authors:** Cristina Delgado-Andrade, Raquel Olías, Ana Haro, M. Carmen Marín-Manzano, Leticia Benavides, Alfonso Clemente, Isabel Seiquer

**Affiliations:** Departamento Nutrición y Producción Animal Sostenible, Estación Experimental del Zaidín, Consejo Superior de Investigaciones Científicas (CSIC), San Miguel 101, Armilla, 18100 Granada, Spain; raquel.olias@eez.csic.es (R.O.); ana.haro@eez.csic.es (A.H.); maricarmen.marin@eez.csic.es (M.C.M.-M.); e.leticiabx@go.ugr.es (L.B.); alfonso.clemente@eez.csic.es (A.C.)

**Keywords:** soy beverages, in vitro gastrointestinal digestion, bioaccessibility, lipid profile, calcium, magnesium, antioxidant action, Caco-2

## Abstract

Soy beverages (SB) are the most popular beverage in the expanding market of plant-based drinks. They provide high-quality protein and polyphenols and are often Ca-fortified as a milk alternative. This work evaluated the antioxidant potential, the mineral content, and the fatty acid profiles found in SB, analysing as well the bioaccessibility of some nutrients after INFOGEST static digestion. Five types of SB available in the market, including Ca-fortified, high-protein, and low-fat drinks, were analysed. Ca supplementation and high protein content in the beverages significantly enhanced Ca bioaccessibility. The lipid profile demonstrated substantial changes during digestion, due to drastic reductions in saturated fatty acids and marked increases in polyunsaturated fatty acids in the bioaccessible fractions; these changes were mainly related to the content levels of Ca and polyphenols in the beverages. Significant increases in the antioxidant properties, as measured by ABTS and FRAP assays, were noticed after the digestive process. Additionally, ROS generation in Caco-2 cells after induced oxidative damage was prevented by the BF of digested SB. The digested low-fat drink, which also had a low level of protein content, showed the least antioxidant activity. In conclusion, composition of the soy drink considerably affected the bioaccessibility of nutritional components and the drink’s antioxidant potential.

## 1. Introduction

The market for plant-based drinks (PBDs) has been experiencing significant global growth, partially displacing the consumption of cow’s milk for various reasons. On one hand, this trend is driven by consumers seeking more sustainable diets, with animal-based products being partially or fully replaced by plant-based alternatives. On the other hand, PBDs are valued for their health benefits, making them a preferred choice for individuals with lactose intolerance or cow’s milk protein allergies. These factors have led to a notable rise in demand for plant-based beverages in recent years [1]. A survey conducted as part of the European Smart Protein Project (https://smartproteinproject.eu/, on 13 February 2025) revealed that, only outranked by Germany, Spain otherwise leads in the consumption of PBDs in Europe, soy beverages being the second most popular choice, after oat-based drinks. From a nutritional perspective, soy-based drinks are the only PBD that provide protein with a biological value equivalent to that of milk or egg protein, but without cholesterol and with low levels of saturated fatty acids [2]. They are rich in vitamin B, unsaturated fatty acids, and beneficial compounds like phytosterols, soy lecithin, and isoflavones. Due to this nutritional profile, various studies have demonstrated soy milk’s protective effects against a range of health conditions, including cancer, osteoporosis, and cardiovascular diseases [3,4,5]. Components such as isoflavones, essential fats, and amino acids have been linked to cardiovascular benefits in both healthy and at-risk populations [6]. Interestingly, combinations of soy proteins, fatty acids, and phytoestrogens appear to be more effective than isolated phytoestrogens or soy proteins alone [7], emphasizing the role of the essential mono- and polyunsaturated fatty acids present in soy beverages for cardiovascular health [6]. While the bioaccessibility of phytoestrogens and proteins after gastrointestinal digestion of soy drinks has been explored [8,9], little attention has been paid to the bioaccessibility of fatty acids and minerals in these beverages. Additionally, the antioxidant properties of bioactive compounds in soy seeds contribute significantly to the biological effects observed with long-term consumption of soy-based drinks. A recent study evaluated the antioxidant capacity of various PBDs (including rice, oat, coconut, hazelnut, spelt, quinoa, tiger nut, and soy) against semi-skimmed cow’s milk (1.5% fat), using in vitro methods like ORAC, ABTS, and FRAP assays. Among the tested beverages, soy drinks demonstrated the highest antioxidant activity after in vitro gastrointestinal digestion, regardless of the assay used [10].

The market now offers a wide variety of soy beverages tailored to different consumer preferences and needs. The review by Olías et al. [1] analyzed 52 commercial soy drinks from 16 brands available in Spain and other European countries. Based on label information, these drinks were categorized into five groups: (i) original soy drinks; (ii) fortified soy drinks, with added calcium (minimum 7.5% of the nutrient reference values); (iii) sugar-free soy drinks, labeled as “no added sugars”; (iv) light soy drinks, with 1.5 g fat/100 mL and 30% fewer calories than the original product; and (v) flavored soy drinks, with added flavors such as chocolate, vanilla, caramel, cappuccino, nuts, cinnamon, or lemon. The inclusion of ingredients to enhance calcium content, improve flavor, or adjust soybean concentration for targeted protein or energy levels introduces changes to the food matrix that may affect nutrient and mineral bioaccessibility.

Given the extensive variety of soy beverages on the market, this study takes a step beyond just analyzing their composition, highlighting the crucial role of antioxidant activity. In addition to assessing the fatty acids and mineral composition of soy drinks formulated for different consumer groups, we focused on their antioxidant potential. By examining the bioaccessibility of fatty acids and key macro-minerals (calcium, magnesium, and potassium), along with antioxidant activity after in vitro gastrointestinal digestion, we provide a deeper understanding of how variations in the food matrix influence both nutrient absorption and functional health benefits.

## 2. Materials and Methods

### 2.1. Reagents and Chemicals

Methanol and chloroform were sourced from VWR (Barcelona, Spain), while sodium bicarbonate, acetate sodium, gallic acid, and hydrochloric acid were obtained from Merck (Darmstad, Germany). The ABTS (2,2′-Azino-bis (3-ethylbenzothiazoline-6-sulfonic acid) diammonium salt was purchased from Amresco (Solon, OH, USA). Fluka Chemicals (Madrid, Spain) provided 2,4,6-Tri(2-pyridyl)-s-triazine (TPTZ) and iron (III) chloride for the ferric reducing antioxidant power (FRAP) assay. Trolox (6-hydroxy-2,5,7,8-tetramethylchromane-2-carboxylic acid), Folin-Ciocalteu reagent, diethyl ether, and enzymes such as pepsin (porcine gastric mucosa, P6887) and pancreatin (porcine pancreas, P7545) were also sourced from Sigma-Aldrich (St. Louis, MO, USA). Bile salts (B3883), 4-(2-hydroxyethyl)-1-piperazineethanesulfonic acid (HEPES), tert-butylhydroperoxide (t-BOOH), cell culture media, cell culture-grade chemicals, fatty acid standards (C15:0 and CRM47885 Supelco 37 Component FAME Mix), and all other reagents used for mineral analysis were likewise obtained from Sigma-Aldrich. All chemicals were of analytical or high purity grade. Bi-distilled deionized water (Milli-Q purification system, Millipore, Bedford, MA, USA) was used throughout.

### 2.2. Samples

Having conducted a previous study of the soy-based beverages available on the market [1], the authors devised an experimental protocol consisting of a comparison between five commercial soy beverages (SB) made exclusively from soybeans, and in the absence of added sugars or flavorings. These beverages represented well the current soy-beverage market in Spain and Europe and are widely consumed. The SB were acquired in local markets, and, for each type of drink, two different batches were analysed. Beverages were codified as SB1, SB2, SB3, SB4, and SB5. SB1, SB2, and SB3 were of different brands, but declared in their labelling the same protein content (30g/L), as well as the lack of added sugars; contrastingly, SB4 declared a high protein content (50 g/L) and SB5 was branded as low fat (“light”) and as having a lower protein content (20g/L). SB1, SB4, and SB5 were options aimed at different segments of the population, variously demanding calcium/protein supplementation or caloric restriction; all of them were from the same commercial company. The specific composition and percentage of soy seeds utilized in drink production are included in Table 1. The five SB were properly homogenized in a common stirrer for 30 min., and then aliquoted in polyethylene containers sealed under vacuum and stored at 4 °C for immediate use in the subsequent analyses and trials.

### 2.3. Nutrient Composition Analysis

All analyses were conducted in triplicate. Gross energy was measured using an isoperibolic bomb calorimeter (Parr Instrument Co., Moline, IL, USA). Total nitrogen was determined by the Dumas procedure using LECO Truspec CN equipment (LECO Corporation, St. Joseph, MI, USA), and protein content was calculated by multiplying the nitrogen value by a factor of 6.38. Mineral content was analyzed following the procedure outlined by Haro et al. [11]. In brief, after wet digestion of the samples (HNO_3_:HClO_4_ 1:4, 180–220 °C), calcium (Ca), magnesium (Mg), sodium (Na), potassium (K), iron (Fe), copper (Cu), and zinc (Zn) were quantified using flame atomic absorption spectroscopy (FAAS) on a Perkin–Elmer Analyst 700 Spectrophotometer (Norwalk, CT, USA). Certified reference standards (European Commission, Reference Materials Unit, Geel, Belgium) were utilized to verify method accuracy. All glassware and polyethylene bottles used for mineral analysis were thoroughly rinsed with 10 mM nitric acid and Milli-Q water.

### 2.4. In Vitro Gastrointestinal Digestion

The simulated digestion was performed, at minimum in triplicate, according to the established INFOGEST method [12,13]. Enzyme activities and bile concentrations were measured according to the assays described previously [12], with the modification described in Sousa et al., [14] for pancreatin suspension. Pancreatin and simulated intestinal fluid (SIF) were mixed, subjected to 5 min of ultrasonic treatment, centrifuged (2000× *g*, 4 °C, 5 min), and the supernatant used for the intestinal phase of digestion. The process consisted of oral, gastric, and intestinal phases.

For the oral phase, 5 g of the beverage was mixed with 5 g of simulated salivary fluid (SSF). Salivary α-amylase was omitted since it is not needed in the case of liquid foods due to the short time spent in the mouth, and the fact that starch is not present in the soy beverages analyzed [13]. The sample was incubated at 37 °C for 2 min with gentle and continuous agitation. During the gastric phase, the oral bolus was combined with simulated gastric fluid (SGF), and the pH was adjusted to 3.0. Porcine pepsin (3359 U/mg) was added to reach a final concentration of 40,000 U in the mixture, which was then immediately incubated in a water bath at 37 °C with gentle shaking then performed for 2 h. After gastric digestion, the pH was adjusted to a pH of 7.0. For the intestinal phase, simulated intestinal fluid (SIF) was added to the mixture, along with 4000 U of pancreatin (5.18 U trypsin/mg) and 160 mM bile solution (B3883; 2.40 mmol of bile salts/g). The digesta was incubated for 2 h in a water bath at 37 °C, with mixing at 80 rpm. To halt the intestinal digestion, the samples were snap-frozen. Upon thawing on ice, the samples were centrifuged at 10,000× *g* at 4 °C for 45 min to separate the bioaccessible fraction (BF) from the residual fraction (RF). A control digestion was performed by replacing the food with Milli-Q water. Aliquots of the BF were stored at −80 °C in light-protected tubes under a nitrogen atmosphere and were later used for antioxidant analyses and cell culture assays. For cell experiments, the BF was filtered through a 30 KDa cutoff filter (Amicon ultra-15 15mL, Millipore). Additionally, the fatty acid profiles and mineral content (Ca, Mg and K) were analyzed in both the BF and the RF.

### 2.5. Antioxidant Activity and Total Phenolic Content

Before measuring antioxidant activity and total phenolic content (TPC), a chemical extraction was carried out on the undigested samples, following the method outlined by Pérez-Jiménez and Saura-Calixto [15]. In brief, 0.250 g of lyophilized beverage was placed in a polypropylene tube, and 6 mL of acidic methanol/water (50:50 *v*/*v*, pH 2) was added. The tube was shaken thoroughly at room temperature for 20 min and then centrifuged at 2500× *g* for 10 min. The supernatant was collected, and a second extraction was performed by adding 4 mL of the same acidic methanol/water added to the residue. The shaking and centrifugation steps were repeated, and the second extract was combined with the first. Extraction was carried out in duplicate. The BF was used directly for analysis. If needed, appropriate dilutions with distilled water were made for the measurements of antioxidant activity and TPC.

The ABTS assay method evaluates the ability to scavenge free radicals and was carried out with, minor modifications, based on the protocol described by Rufián-Henares and Delgado-Andrade [16]. To generate the ABTS+· radical, a 7 mM ABTS stock solution was mixed with 2.45 mM potassium persulfate and allowed to incubate in the dark at room temperature for 12–16 h. The resulting ABTS+· working solution, which is stable for 2 days, was diluted with an ethanol/water (50:50) mixture to achieve an absorbance of 0.70 ± 0.02 at 730 nm. For the analyses, 40 µL of the sample (chemical extract or BF), blank, or Trolox standard and 200 µL of 5 mM phosphate buffer (pH 8.4) were added to 60 µL of the diluted ABTS+· solution. Absorbance was measured after 10 min using the previously described microplate reader. Aqueous Trolox solutions (15–125 µM) were used to create the calibration curve. Results were determined as µmol of Trolox equivalents (TE)/mL of the beverage. All measurements were performed in triplicate.

The FRAP assay measures the ferric reducing antioxidant power (FRAP) and was carried out following the method described by Seiquer et al. [17]. The FRAP reagent was freshly prepared each day by combining 10 mM Fe^2+^-2,4,6-Tri(2-pyridyl)-1,3,5-triazine (TPTZ), 40 mM HCl, 20 mM ferric chloride, and 0.3 M sodium acetate buffer (pH 3.6) at a 1:1:10 *v*/*v*/*v* ratio. For the analysis, 20 µL of the sample (chemical extract or BF), blank, or Trolox standard were mixed with 280 µL of pre-warmed FRAP reagent (37 °C) and incubated at 37 °C for 30 min in the dark. Absorbance was measured at 595 nm. Aqueous Trolox solutions, as mentioned earlier, were used for calibration. The results are expressed as µmol of Trolox equivalents (TE)/mL of the beverage. All measurements were conducted in triplicate.

The total phenolic content (TPC) was measured using the Folin–Ciocalteau colorimetric method [17]. To perform the analysis, 10 µL of the sample (chemical extract or BF), blank, or gallic acid standard, along with 10 µL of Folin–Ciocalteau reagent, were mixed and left to stand for 3 min. Next, 200 µL of a sodium carbonate solution (75 g/L) was added, and the volume adjusted to 250 µL with Milli-Q water. The mixture was then shaken and allowed to incubate in the dark for 60 min. Absorbance was measured at 750 nm, and results were quantified using a gallic acid standard curve (25–250 mg/L). The TPC is expressed as µmol of gallic acid equivalents (GAE)/mL of the beverage. All measurements were conducted in triplicate.

### 2.6. Reactive Oxygen Species (ROS) Generation in Caco-2 Cells

The BFs obtained after in vitro digestion of the soy beverages were tested to evaluate their antioxidant potential at the cellular level by assessing the effects on the reactive oxygen species (ROS) generation in Caco-2 cells, following the method outlined by Borges et al. [18].

Caco-2 cells were obtained from the European Collection of Cell Cultures (ECACC) through the Cell Bank of Granada University (Granada, Spain). The cells were cultured in 75 cm^2^ plastic flasks (Costar, Cambridge, MA, USA) and maintained through serial passaging. The culture medium consisted of high-glucose Dulbecco’s modified minimal essential medium (DMEM), supplemented with 10% heat-inactivated fetal bovine serum (FBS), 3.7 g/L NaHCO_3_, 1% non-essential amino acids, 15 mM HEPES, 0.1 UI/mL bovine insulin, and 1% antibiotic–antimycotic solution. ROS production was measured under basal conditions and following induction of oxidative stress. The experiments were conducted using a mixture of BF and FBS-free DMEM (1:1 *v*/*v*), a ratio chosen based on prior tests using the MTT assay (3-(4,5-dimethylthiazol-2-yl)-2,5-diphenyltetrazolium bromide, Roche, Mannheim, Germany), which confirmed that cell viability remained above 90% under these conditions.

ROS generation was assessed using the dichlorofluorescin (DCFH) assay. In brief, Caco-2 cells were plated in 24-well plates at a density of 10 × 10^4^ cells/mL (400 µL/well) and allowed to grow for 48 h. After removing the spent medium, cells were pre-incubated with the BF for 2 h. Next, the cells were treated with 20 µM DCFH and incubated for 1 h. The DCFH solution was then removed, and either fresh culture medium (for basal measurements) or 20 mM t-BOOH (to induce oxidative stress) was added. The fluorescence intensity was measured at excitation and emission wavelengths of 485 nm and 535 nm, respectively, over a period of 0–90 min at 37 °C. In the presence of free radicals such as ROS, the DCFH is oxidized into dichlorofluorescein (DCF), which emits fluorescence, enabling the quantification of ROS production. The results describing ROS generation are expressed as fluorescence units.

### 2.7. Fatty Acid (FA) Analysis

FA analysis was conducted both before and after the in vitro digestion of the soy beverages. For the analysis, 10 mL aliquots of the BF were lyophilized in triplicate using a LyoQuest-85 freeze-dryer (Telstar, Terrasa, Spain).

Fat was extracted using a 2:1 chloroform/methanol mixture (*v*/*v*) [19], and the extracted FA were methylated following the method of Kramer et al. [20], with minor modifications. The lipid fraction was first methylated with NaOH/methanol (50 °C, 15 min); this was followed by methylation with HCl/methanol (50 °C, 1 h) to obtain fatty acid methyl esters (FAME). Pentadecanoic acid (C15:0) was used as an internal standard. The fatty acid profile was analyzed using a gas chromatograph (Focus GC, Thermo Scientific, Milan, Italy) equipped with a split/splitless injector, a flame ionization detector, and a 100 m × 0.25 mm × 0.2 µm capillary silica gel column (SP 2560 Supelco, Bellefonte, PA, USA). The temperature of the program ranged from 70 to 240 °C, with the injector and detector set at 250 °C. Helium was used as the carrier gas at a flow rate of 1 mL/min. Individual FAME peaks were identified by comparing their retention times with known standards (Supelco 37 Component FAME Mix). The results are expressed as the relative percentage of each fatty acid of the total identified, as calculated by internal normalization of the chromatographic peak area.

### 2.8. Statistical Analysis

Statistical analyses were conducted using SPSS version 16.0 (SPSS Inc., Chicago, IL, USA) and Statgraphics Centurion XV (Herndon, VA, USA). To assess the significance of the variables, a one-way analysis of variance (ANOVA) was performed, followed by mean comparisons using the HSD Tukey test. Pearson’s linear correlation coefficient was calculated to examine the relationships between different variables. All statistical tests were considered significant at a *p*-value of less than 0.05.

## 3. Results and Discussion

### 3.1. Nutritional Composition of the Selected Soy Beverages

The nutritional composition determinations for the commercial soy beverages (SB) are summarized in Table 1 and briefly described here. The SB selected for this study were from three different brands and have varying proportions of soybeans (8.7–14.5%), likely reflecting differences in origins, ecotypes, and/or agronomic conditions. This variability is evident in the lack of correlation between the soybean content and the protein levels in the beverages. For instance, SB4, which had the highest protein concentration, contained nearly the same soybean proportion per 100 mL as SB2 and SB3. However, the protein content values in SB2 and SB3 were 42% and 26% lower, respectively, than that of SB4. Additionally, although SB5, the low-fat option, presented the lowest proportion of soybeans, its energy supply was relatively similar to those of SB1 and SB2 (30.5 vs. 38.1 and 35.5 kcal/100 mL, respectively). SB5 exhibited lower levels of most macro- and micronutrients, with the exception of Ca- which was fortified using an external source, as indicated on the label, to match the Ca supply of traditional cow’s milk- Na, P, and carbohydrates. Ca supplementation was as well evident in SB1 and SB4, as these three beverages (SB1, SB4, and SB5) belonged to the same commercial brand. Among all samples, SB4 showed the most complete nutrient profile. Based on the overall composition of SB studied, when Ca is not supplemented, these drinks primarily serve as sources of Na, Mg, K, P, and protein. It is worth noting that only SB1, SB4, and SB5 declared their vitamin contents on the labels, with no differences reported among them. The nutritional data align with the findings of Olías et al. [1], who analyzed the composition of 52 commercial SB products from 16 brands available in Spain and other European countries.

### 3.2. Calcium, Magnesium, and Potassium Bioaccessibility

Assessing mineral bioaccessibility is essential for determining the nutritional value of soy beverages, particularly when used as alternatives to cow’s milk. Milk is traditionally the primary dietary source of bioavailable calcium, a crucial mineral that, along with magnesium and potassium, plays a vital role in achieving optimal peak bone mass and preventing osteoporosis [21,22,23]. Substituting for cow’s milk with PBDs could have significant implications for bone health, making it critical to evaluate not only the latter’s mineral content but also the extent to which these minerals are bioaccessible and available for absorption.

Despite its importance, mineral bioaccessibility in soy beverages has scarcely been studied. Table 2 presents the bioaccessibility of Ca, Mg, and K following in vitro digestion of the selected soy drinks. Our results indicate that calcium supplementation (using calcium carbonate) in soy beverages SB1, SB4, and SB5 improved this mineral’s bioaccessibility, with levels ranging from 44% to 51%. These values were significantly higher than those observed in the non-fortified soy drinks SB2 and SB3, which exhibited bioaccessibility values between 23% and 27%. This finding underscores the determination that the naturally occurring calcium in soy beverages is poorly bioaccessible and insufficient to meet nutritional requirements. On the other hand, in fortified soy beverages, calcium availability was comparable to that of absorbable calcium from milk [24]. The remaining calcium was detected in the residual fraction (RF), with insoluble calcium proportions ranging from 53% in SB4 to 76% in SB2.

A strong correlation was observed between initial calcium content and soluble calcium after digestion, both in absolute quantity (*r* = 0.9788, *p* = 0.0000) and percentage (*r* = 0.8535, *p* = 0.001). Additionally, protein content influenced calcium bioaccessibility. Among soy beverages with similar calcium levels, those with higher protein content exhibited greater calcium bioaccessibility (51.3% in SB4 vs. 43.8% in SB5), supporting the positive role of dietary protein in calcium availability [21]. These findings emphasize the need for comprehensive analysis of mineral bioaccessibility in plant-based dairy alternatives to ensure their nutritional adequacy and potential benefits for bone health.

The results of the present study regarding fortified drinks showed Ca bioaccessibility values similar to those reported by Siqueira Silva et al. [25], who found figures as high as 45.9% in a comparative study of several PBDs. In their study, a soy drink exhibited the highest bioaccessibility compared to rice, almond, peanut, oat, and coconut beverages. In contrast, Theodoropoulos et al. [26], using a combined methodology of in vitro enzymatic solubilization and dialysis, reported much lower values, ranging from 3.3% to 5.4%. Recently, Muleya et al. [24] studied the bioaccessible calcium in various plant-based products fortified with calcium phosphate using the same INFOGEST procedure as our study. They reported significantly lower Ca bioaccessibility values of 3–5% for all tested drinks, which contrasts with our findings. This suggests that Ca bioaccessibility depends not only on the food matrix’s chemical composition (particularly its phytate and oxalate content) but also on the chemical form of the fortifier. In this regard, both in vitro and in vivo studies have demonstrated that calcium carbonate, the fortifier used in the drinks under study, exhibited higher bioaccessibility than tricalcium phosphate due to its greater solubility under physiological digestion conditions [24,25]. As a result, calcium carbonate leads to Ca bioavailability comparable to that of cow’s milk [27].

Magnesium (Mg) is an essential mineral involved in critical physiological processes, such as blood sugar regulation and muscle contraction, in addition to its role in bone health [22]. Maintaining optimal levels of both Mg and Ca is crucial for overall health, as these divalent minerals may compete at digestive and absorptive levels [28]. The Mg content values among the soy-beverage samples varied significantly, ranging from 10.6 mg/100 mL in SB5 (low fat) to 23.1 mg/100 mL in SB4 (high protein). SB5 also had the lowest soluble Mg after in vitro digestion, nearly half that of SB3 and SB4, which depicted the highest values. However, no significant correlation was found between initial Mg content and its bioaccessibility. In this study, Mg bioaccessibility was found to be negatively correlated with both Ca content (*r* = −0.5689, *p* = 0.0269) and Ca bioaccessibility (*r* = −0.6602, *p* = 0.0074), highlighting the complex interrelations between these minerals [28]. Although the percentage of Mg bioaccessibility did not significantly vary between the soy drinks studied, samples SB2 and SB3, both with low Ca content (mainly because they are not fortified), showed the highest values for bioaccessible Mg and the lowest residual Mg. These findings emphasize the need to consider not only total mineral content but also interactions with other nutrients when assessing mineral bioavailability in plant-based beverages.

Potassium (K) is an essential mineral primarily known for maintaining electrolyte balance and cardiovascular system. However, its crucial role in bone health has also been highlighted [23]. Therefore, maintaining adequate potassium intake can be beneficial in the prevention of cardiovascular disease and osteoporosis, making the consumption of soy drinks a valuable dietary recommendation.

Just like in cow’s milk, K was the most abundant mineral in soy drinks, reaching up to 250 mg/100mL in samples SB1 and SB4. The lowest value (112 mg/100 mL) was found in SB5 (low fat), whereas the non-Ca-fortified drinks, SB2 and SB3, showed content values around 155 mg K/100 mL. The K content was not significantly related to the quantity of soybeans added. However, as a plant-based product, its mineral content can vary according to factors such as practices of cultivation, soil composition, postharvest handling, or use of fertilizers [29] and also with respect to conditions of beverage production [30]. K bioaccessibility was found to be very high in the soy drinks, ranging from 78% in sample SB5 to around 100% in the remaining beverages, and positive correlations between K content and bioaccessibility were observed (*r =* 0.7036; *p* = 0.0034). Our results agree with the scarce reports found in the literature, which show values of K bioaccessibility in soy drinks near of 100% [30].

### 3.3. The Lipid Fraction

Lipid content in soy beverages is influenced primarily by the amounts of soybeans used in production, as soybeans naturally contain 18–24% total lipids. However, other factors such as processing methods and formulation also play a role. In this study, fat content ranged from 1.2 g/100 mL in SB5 (low fat) to 2.8 g/100 mL in SB4 (high protein), values consistent with those reported in the literature [1].

**Figure 1:**
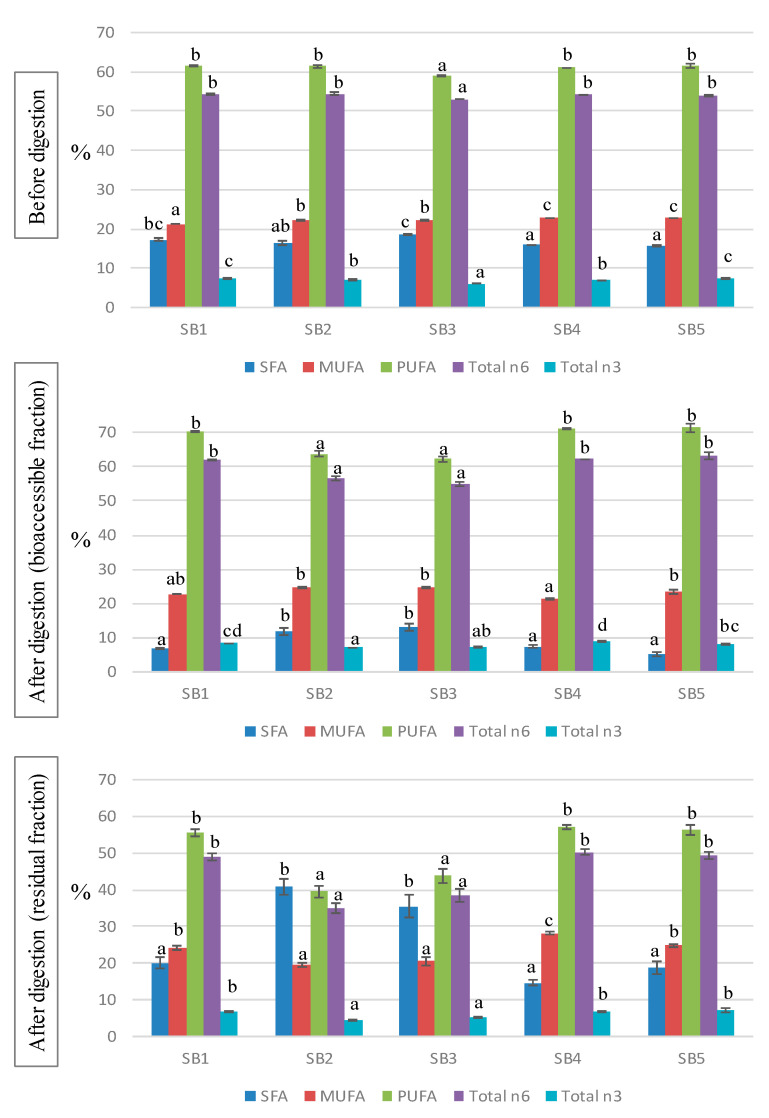
Fatty acid indices before and after the in vitro digestion of the soy beverage. SFA: saturated fatty acids; MUFA: monounsaturated fatty acids; PUFA: polyunsaturated fatty acids. Different letters indicate significant differences between beverages (*p* < 0.05). One-way ANOVA followed by Tukey HDS test.

The fatty acid (FA) profile of the soy beverages is shown in Table 3 and the main indices are represented in Figure 1. The majority of the FAs were polyunsaturated FAs (PUFA), with an average contribution of 61%, whereas monounsaturated FAs (MUFA) and saturated FAs (SFA) accounted for around 22% and 17% of the total FAs, with slight significant differences found between samples. Among PUFA, approximately 90% were n6 and 10% n3 FA. The most abundant FA was linoleic acid (C18:2n6), contributing 53–54% to the total, followed by oleic acid (C18:1n9, 20–21%), palmitic acid (C16:0, 10–12%), and linolenic acid (C18:3n3, 6–7%). The FA profiles of the studied soy beverages are in accordance with previous studies [1,9,31]. The particular FA profile of soy drinks, rich in unsaturated FAs and containing only a small amount of SFAs, is considered to have positive health effects particularly due to the associated preventive action with respect to cardiovascular diseases [32].

The composition of FAs was analyzed as well after the in vitro digestion of the soy drinks, both in the BF and the RF (Table 4); these results represent new information in the literature. During digestion, the FA profiles of the samples experienced notable changes that led to different patterns, compared with those of the original beverages. In the bioaccessible fraction, a great decrease in SFA was observed, representing from 5 to 13% of the total, whereas MUFA and, especially, PUFA increased to average values of 23% and 68%, respectively. The main modifications in the BF were due to the drop in palmitic acid and the rise in linolenic acid. On the contrary, the residual fraction (usually discarded in bioavailability studies) resulted in enriched SFA (20–40%) and lower contributions of PUFA (40–57%), compared with undigested samples. The high SFA content values in the residual fractions warrant further research to understand its possible impact on the intestinal microbiota.

There is a lack of information concerning the FA profiles of soy drinks after the digestive process. García-Casas et al. [33] showed significant changes in the fat composition during the digestion of whey-based beverages, based on drastic reductions in SFA and marked increases in MUFA and PUFA, in line with our results. The decline in SFA may be attributed to the formation of insoluble soaps between Ca and saturated long chain FA (<14C), reducing their availability in the BF [34]. Accordingly, bioaccessible SFA levels were lower in beverages supplemented with Ca (SB1, SB4 and SB5) than in the non-fortified drinks (SB2 and SB3). The reduction in soluble SFA was mainly due to the precipitation of palmitic and stearic acids, which, in turn, increased in the RF. The digestion process also induces oxidation of PUFA [35], although the use of antioxidants such polyphenols and, particularly, catequines, may avoid lipid oxidation, thus promoting the bioavailability of non-oxidized PUFA [36]. Additionally, the oxidation pattern depends on the food matrix, and emulsified lipids were found to be better protected than non-emulsified lipids [37]. Therefore, the high levels of polyphenols in the soy beverages, combined with the fact that lipids in soy drinks are stored in little spherical organelles called oleosomes acting as a natural emulsion [1], prevented lipid oxidation, leading to an increased concentration of PUFA in the BF.

### 3.4. Total Phenolic Compounds and Antioxidant Properties: Effects of In Vitro Digestion

Soy beverage is a source of different phytochemicals such us isoflavones and phenolic acids (gallic acid, 4-hydroxybenzoic acid, p-coumaric acid, ferulic acid, naringenin, rutin, quercetin, and (+)-catechin) with antioxidant action [38]. Isoflavones are well recognized for their protective effects against some important degenerative diseases like cancer, atherosclerosis, and osteoporosis [39]. However, the real in vivo effects of these compounds would be determined by their bioaccessibility after gastrointestinal digestion. To come closer to the in vivo situation, in the present work, the TPC and the antioxidant activity of soy beverages were evaluated before and after the in vitro gastrointestinal digestion (Table 5). Statistical differences regarding the TPC in the undigested beverages were found; standout results included the higher content detected in SB4 (high protein).

A positive significant correlation was identified between the protein content values of samples and the TPC (*r =* 0.8451; *p* = 0.0021). The results were consistent with data reported by Ma et al. [40] for different heat-treated soy milks. After in vitro digestion, the values increased by a factor of 15–20 times, with BS5, the light drink, being the sample with the lowest TPC (*p* < 0.05). Rodríguez-Roque et al. [38] analysed the concentration of phenolic compounds during in vitro digestion of soy milk and also established that there were greater amounts in the digested samples when the Folin-Ciocalteu method was used, but the increase was lower than in the present assay. Saura-Calixto, Serrano, and Goñi [41] reported that phenols linked to high molecular weight compounds, such as proteins and carbohydrates, may be released by digestive enzyme action, leading to a significant increase in their concentrations after gastric digestion. It has been also shown that digestive enzymes and pH conditions improve the release of phenolic compounds from the soy drink matrix by converting the polymeric polyphenols to monomeric compounds, thus increasing the TPC in the digested sample [10]. In this sense, heat-treated soy drinks favoured antioxidant activity after digestion due to increases in phenolic and flavonoid contents in the digested fraction [40].

Since the measurement of the antioxidant activity is dependent on the reaction mechanism, a single method cannot be used for the evaluation of all phytochemicals in the extract. A combination of several tests is recommended as a more reliable assessment of the antioxidant profile [42]. In the present study, two chemically based antioxidant analyses were conducted: ABTS, which tests the free radical scavenger ability; and FRAP, which evaluates the antioxidant action through the ferric reducing capacity. In the commercial samples, ABTS values ranged from 0.74 to 2.53 µmol TE/mL drink, whereas FRAP oscillated between 0.37 to 0.65 µmol TE/mL drink; SB5 (low fat) and SB4 (high protein) returned the lowest and highest values for these parameters, respectively. Previous studies reported that the ABTS and FRAP values associated with soy drinks are the highest among different PBDs, such as rice, oat, coconut, or hazelnut [10]. In our research, significant relationships were observed using both antioxidant measurements with the protein content values of the undigested drinks (*r* = 0.9224 and *r* = 0.9269 for ABTS and FRAP, respectively, *p* = 0.0001 for both). In addition, TPC was also correlated with the ABTS (*r* = 0.6615, *p* = 0.0072) and FRAP values (*r* = 0.7587, *p* = 0.0010), which is consistent with previous studies performed in extracts of soy beverages [43]. The recent investigation by Aly et al. [10] has established an ABTS value for soy beverages similar to that measured in the present study (2.09 µmol TE/mL drink), but our FRAP values are closer to the figures they mentioned for other plant-based milk substitutes (0.14–0.77 µmol TE/mL drink) and to the level stated by Subrota et al. [44] (0.72–0.80 µmol TE/mL drink). Contrary to findings established by Rodríguez-Roque et al. [38], but in agreement with others [10], after gastrointestinal digestion, all soy drinks increased their antioxidant action, although to a lesser extent than the TPC. In this sense, it is worthy to mention that the Folin-Ciocalteu method is simple and useful in assessing the overall phenolic content, but other non-phenolic substances in food, such as ascorbic acid, sugars, aromatic amines, organic acids, and proteins, can be also reduced by the Folin reagent, thus leading to some overestimation of the phenolic content [45]. The ABST values of digested drinks did not show significant differences between them, and in the FRAP test only the BS5 sample exhibited a significantly lower activity than the rest, with the SB4 returning the highest value. The data are in the same order of magnitude of those reported by Aly et al. [10] for both antioxidant markers, and double that described by Ma et al. [40] for FRAP. Interestingly, after digestion, the TPC values of the soy drinks were strongly related with the FRAP values (*r* = 0.8911, *p* = 0.0000), but not with the ABTS results, suggesting that during the digestive process, other compounds with scavenger properties against free radicals are formed. Similar changes in relationships after digestion were previously observed in olive oil samples by Borges et al. [46]. In addition, peptides derived from digested soy protein showed significant antioxidant capacity, as measured by DPPH and ABTS assays, especially those with molecular weight <3 kDa [47].

### 3.5. ROS Generation in Caco-2 Cells

**Figure 2:**
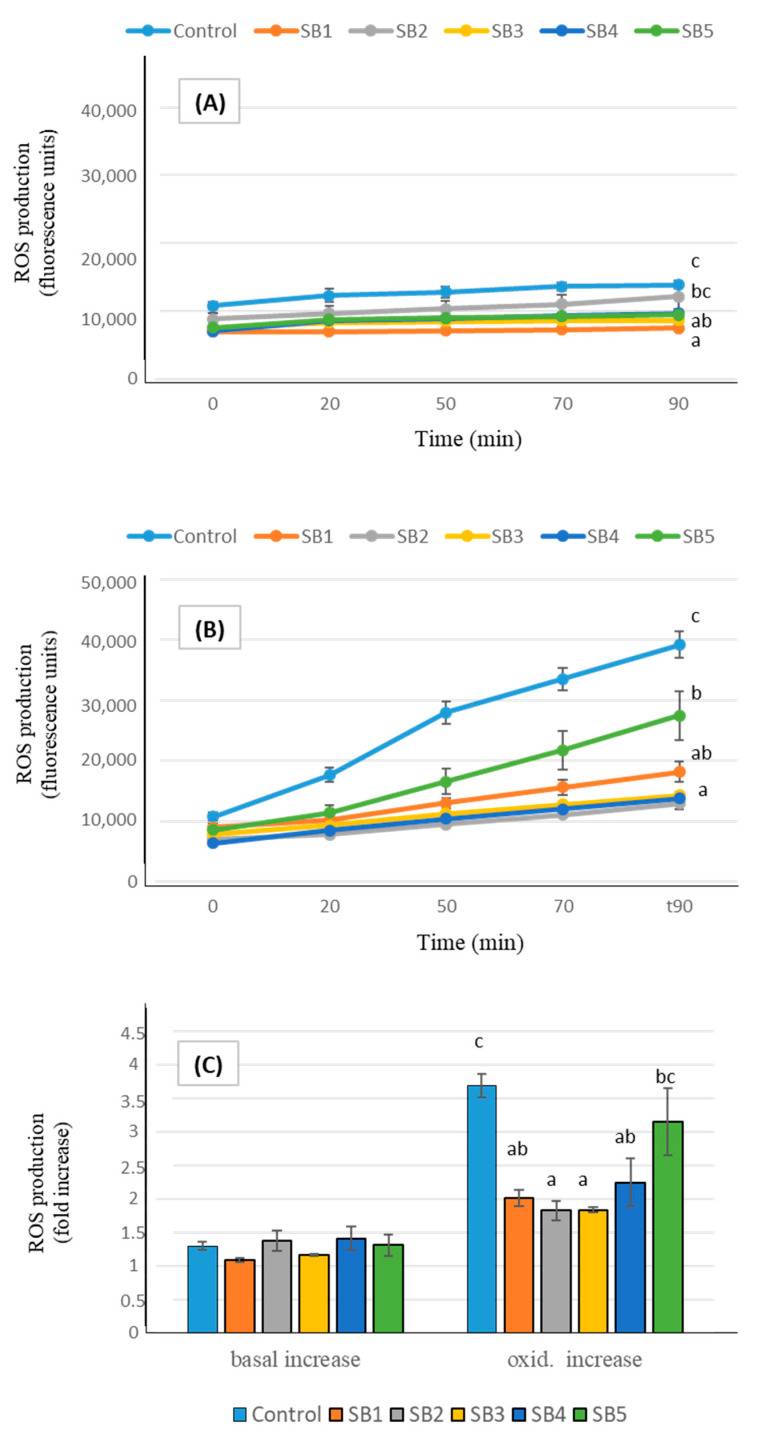
ROS generation expressed as fluorescence units during 90 min (mean ± SE), in Caco-2 cells pre-incubated with the BF of the soy beverages, compared with control cells. Different letters indicate significant differences between beverages (*p* < 0.05). One-way ANOVA followed by Tukey HDS test. (**A**): basal conditions; (**B**): oxidative stress induced with t-BOOH 20 mM; (**C**): total increase in ROS expressed as fold number.

In addition to in vitro analyses, studies of antioxidant cell markers enabled the elucidation of the in vivo antioxidant impacts of foods on human health [46]. The ROS generation by Caco-2 cells after exposure to the digested soy drink samples is shown in Figure 2. Under basal conditions (A), cells experienced a slight ROS production, in consequence of their normal functionality, and, although final levels were significantly different from controls after incubation with some of the soy beverages, increases in ROS production during the 90 min (expressed as n-fold increase, part C of the figure) did not differ between samples. Exposing the cells to an oxidative injury by treatment with an oxidizing agent caused a drastic increase in ROS generation by cells (B). In such conditions, pre-incubation with the BF of the soy drinks prevented the oxidative damage, causing decreases in ROS levels between 30% (sample SB5, low fat) and more than 60% (SB2, SB3, and SB4), compared with control oxidized cells. The global ROS increase effected by Caco-2 cells under oxidative conditions (C) was lower than in controls after exposure to all the soy samples; although the low fat beverage (SB5) presented the highest ROS increase among the samples, with no significant difference relative to controls, it still retained a moderate protective effect against oxidation.

Antioxidant markers of soy drinks at the cellular level have scarcely been studied. Aresta et al. [32] reported that both animal and plant-based undigested milks (including soy) reduce ROS production in Caco-2 cells under basal conditions, compared to control cells. However, after pro-oxidant stimulation, only animal milks retained this ability. In contrast, Sun et al. [48] observed a significant decrease in ROS content in oxidative stress-induced Caco-2 cells following incubation with soy drink. A key difference between these studies and our approach is that we used digested samples, which provide a more physiologically relevant assessment of the antioxidant capacity, as digestion can significantly alter the bioactivity of dietary compounds. Several compounds in SB may contribute to their antioxidant effects, as both soy isoflavones and soybean proteins have been shown to be associated with active antioxidant actions in cultured Caco-2 cells [49,50]. While the antioxidant capacity of SB has largely been attributed to their non-lipid components, the potential contribution of the lipid fraction should not be overlooked. The antioxidant properties of the lipid fraction in plant-based milks have been rarely investigated, yet Aresta et al. [32] found that, despite the primary scavenging activity being associated with the non-lipid fraction, the lipid fractions of soy drinks still retained significant antioxidant potential. Specifically, it was able to reduce ROS production in Caco-2 cells following induced oxidation. Given that digestion can modify the bioactivity of lipids, further research is needed to elucidate the specific role of the lipid fraction in the antioxidant properties of soy drinks post-digestion.

## 4. Conclusions

This study highlights the influence of soy-beverage composition on the bioaccessibility of essential minerals, fatty acids, and antioxidant properties. Calcium fortification and protein content were identified as key factors enhancing mineral bioaccessibility, particularly for calcium, which reached levels comparable to those found in cow’s milk in fortified beverages. It is important to recognize that the methodologies used to determine total phenolic content and antioxidant capacity have inherent technical limitations. While informative, they do not fully capture the complexities of antioxidant mechanisms or identify all contributing compounds. Nevertheless, it is worth mentioning that the in vitro gastrointestinal digestion process led to an increase in total phenolic compounds and antioxidant activity, with variations observed among different soy-beverage formulations. Additionally, soy drinks displayed beneficial lipid profiles, characterized by low SFA levels and marked increases in PUFA following in vitro digestion. Further studies, including more comprehensive assessments of antiradical activity and the identification of specific antioxidant compounds, are necessary to confirm and expand upon these findings.

## Figures and Tables

**Table 1:** Nutritional composition of soy beverages (per 100 mL drink).

	SB1	SB2	SB3	SB4	SB5
Soybeans (g)	8.7	14.0	14.5	13.1	5.6
Energy * (kcal)	38.1 ± 0.1 ^c^	35.5 ± 0.1 ^b^	44.2 ± 0.1 ^d^	62.8 ± 0.1 ^e^	30.5 ± 0.1 ^a^
Fats (g)	1.8	1.7	1.9	2.8	1.2
Saturated	0.3	0.3	0.3	0.5	0.2
Monosaturated	0.4	-	-	-	-
Polysaturated	1.1	-	-	-	-
Carbohydrates (g)	0	1.3	1.6	2.5	1.7
Sugars	0	0.5	0.8	2.5	1.5
Fiber (g)	0.6	-	<0.5	0.9	0.9
Protein * (g)	3.3 ± 0.1 ^c^	2.9 ± 0.1 ^b^	3.7 ± 0.1 ^d^	5.0 ± 0.1 ^e^	2.1 ± 0.1 ^a^
Minerals * (mg)					
Ca	129.0 ± 0.6 ^b^	12.2 ± 0.1 ^a^	16.7 ± 0.1 ^a^	134.0 ± 2.2 ^b^	122.0 ± 5.4 ^b^
P	112.0 ± 0.4 ^b^	45.9 ± 0.8 ^a^	50.6 ± 0.1 ^a^	136.0 ± 2.4 ^c^	50.7 ± 0.8 ^a^
Mg	15.5 ± 0.1 ^b^	17.7 ± 0.2 ^c^	20.4 ± 0.1 ^d^	23.1 ± 0.1 ^e^	10.6 ± 0.1 ^a^
Na	23.2 ± 1.0 ^a^	50.9 ± 0.5 ^c^	24.2 ± 0.8 ^a^	36.1 ± 0.5 ^b^	34.1 ± 0.2 ^b^
K	252.0 ± 1.5 ^d^	149.0 ± 0.5 ^b^	161.0 ± 1.6 ^c^	255.0 ± 3.1 ^d^	112.0 ± 1.2 ^a^
Fe	0.41 ± 0.01 ^b^	0.50 ± 0.04 ^d^	0.51 ± 0.01 ^e^	0.52 ± 0.01 ^b^	0.18 ± 0.01 ^a^
Cu	0.12 ± 0.01 ^c^	0.13 ± 0.01 ^d^	0.10 ± 0.01 ^b^	0.15 ± 0.01 ^e^	0.08 ± 0.01 ^a^
Zn	0.29 ± 0.01 ^b^	0.49 ± 0.01 ^d^	0.39 ± 0.01 ^c^	0.62 ± 0.01 ^e^	0.21 ± 0.01 ^a^
Vitamins					
Vitamin D (µg)	0.75	-	-	0.75	0.75
Vitamin B2 (mg)	0.21	-	-	0.21	0.21
Vitamin B12 (µg)	0.38	-	-	0.38	0.38

Hyphen indicates that the data are not declared in the labelling. An * indicates data analyzed in our laboratory. Values are mean ± SD (*n* = 3). Mean values in each row associated with different letters indicate significant differences between samples (one-way ANOVA and Tukey HDS test, *p* < 0.05).

**Table 2:** Bioaccessibility of Ca, Mg, and K after in vitro digestion of the soy beverages.

	SB1	SB2	SB3	SB4	SB5
	Calcium				
BF (µg/mL)	608 ± 33 ^bc^	27.5 ± 8.75 ^a^	44.9 ± 7.91 ^a^	687 ± 5 ^c^	535 ± 52 ^b^
(%)	47.1 ± 2.59 ^bc^	22.6 ± 7.20 ^a^	26.9 ± 4.74 ^ab^	51.3 ± 0.37 ^c^	43.8 ± 4.28 ^bc^
RF (µg/mL)	718 ± 27 ^b^	92.4 ± 10.0 ^a^	113 ± 7 ^a^	716 ± 7 ^b^	679 ± 51 ^b^
(%)	55.6 ± 2.08 ^ab^	75.8 ± 8.25 ^b^	67.7 ± 4.30 ^ab^	53.5 ± 0.55 ^a^	55.6 ± 4.25 ^ab^
Recovery (%)	103 ± 2.90	98.3 ± 1.20	94.6 ± 5.68	105 ± 0.27	99.4 ± 6.93
	Magnesium				
BF (µg/mL)	134 ± 5 ^b^	170 ± 11 ^c^	201 ± 7.21 ^c^	193 ± 3.21 ^c^	101 ± 5.57 ^a^
(%)	86.2 ± 3.42	96.2 ± 6.34	98.7 ± 3.57	83.5 ± 1.38	95.2 ± 5.29
RF (µg/mL)	40.0 ± 0.48 ^b^	4.78 ± 0.10 ^a^	5.91 ± 0.98 ^a^	55.1 ± 1.44 ^b^	24.1 ± 0.87 ^b^
(%)	25.8 ± 0.31 ^c^	2.70 ± 0.06 ^a^	2.91 ± 0.48 ^a^	23.9 ± 0.63 ^b^	22.8 ± 0.82 ^b^
Recovery (%)	112 ± 3.74	98.9 ± 6.31	102 ± 3.33	107 ± 0.75	117 ± 5.60
	Potassium				
BF (µg/mL)	2609 ± 113 ^d^	1291 ± 23 ^b^	1633 ± 134 ^c^	2659 ± 110 ^d^	868 ± 64 ^a^
(%)	103 ± 5 ^bc^	86.6 ± 1.58 ^ab^	102 ± 8 ^bc^	104 ± 4 ^c^	77.6 ± 5.76 ^a^
RF (µg/mL)	103 ± 2	27.5 ± 1.71	32.2 ± 0.62	151 ± 6	67.0 ± 2.14
(%)	4.06 ± 0.10 ^b^	1.85 ± 0.12 ^a^	2.01 ± 0.04 ^a^	5.92 ± 0.25 ^c^	5.99 ± 0.19 ^c^
Recovery (%)	107 ± 5	88.5 ± 1.46	104 ± 8	110 ± 4	83.6 ± 5.86

BF: bioaccessible fraction; RF: residual fraction. The percentages (%) of BF and RF were calculated based on the initial quantities present in the beverages. Values are mean ± SE (*n* = 3). Mean values in each row that are associated with different letters indicate significant differences between samples (one-way ANOVA and Tukey HDS test, *p* < 0.05).

**Table 3:** Fatty acid profile (%) of soy beverages before the in vitro digestion.

Fatty Acid	SB1	SB2	SB3	SB4	SB5
C10:0	0.06 ± 0.02 ^ab^	0.03 ± 0.001 ^a^	0.03 ± 0.001 ^a^	0.03 ± 0.003 ^a^	0.10 ± 0.007 ^b^
C12:0	0.006 ± 0.001	0.010 ± 0.002	0.008 ± 0.001	0.006 ± 0.001	0.011 ± 0.002
C14:0	0.08 ± 0.005	0.08 ± 0.02	0.09 ± 0.001	0.07 ± 0.001	0.08 ± 0.008
C16:0	11.8 ± 0.21 ^b^	9.98 ± 0.28 ^a^	11.4 ± 0.02 ^b^	11.04 ± 0.02 ^b^	9.81 ± 0.07 ^a^
C16:1	0.07 ± 0.003	0.07 ± 0.005	0.06 ± 0.001	0.07 ± 0.001	0.07 ± 0.005
C18:0	4.27 ± 0.01 ^ab^	5.32 ± 0.27 ^c^	6.16 ± 0.03 ^d^	3.77 ± 0.01 ^a^	4.72 ± 0.02 ^b^
C18:1 n9	19.8 ± 0.07 ^a^	20.8 ± 0.17 ^b^	21.0 ± 0.15 ^bc^	21.5 ± 0.04 ^c^	21.4 ± 0.07 ^c^
C18:1 n7	1.14 ± 0.04	0.98 ± 0.09	0.98 ± 0.03	1.17 ± 0.05	1.10 ± 0.01
C18:2 n6	54.0 ± 0.18 ^b^	54.3 ± 0.35 ^b^	52.8 ± 0.07 ^a^	54.0 ± 0.02 ^b^	53.8 ± 0.17 ^b^
C18:3 n6	0.02 ± 0.003	0.03 ± 0.005	0.02 ± 0.001	0.02 ± 0.003	0.03 ± 0.001
C18:3 n3	7.21 ± 0.06 ^c^	6.89 ± 0.08 ^b^	6.00 ± 0.02 ^a^	6.86 ± 0.01 ^b^	7.36 ± 0.10 ^c^
C20:0	0.39 ± 0.02	0.39 ± 0.02	0.32 ± 0.03	0.34 ± 0.01	0.33 ± 0.01
C20:3 n3	0.01 ± 0.001 ^a^	0.01 ± 0.001 ^a^	0.01 ± 0.001 ^a^	0.01 ± 0.001 ^a^	0.03 ± 0.005 ^b^
C20:4 n6	0.05 ± 0.001	0.05 ± 0.003	0.05 ± 0.001	0.06 ± 0.001	0.05 ± 0.001
C20:5 n3	0.09 ± 0.01 ^c^	0.07 ± 0.001 ^b^	0.09 ± 0.006 ^c^	0.06 ± 0.001 ^b^	0.04 ± 0.001 ^a^
C22:0	0.42 ± 0.005 ^b^	0.37 ± 0.02 ^a^	0.35 ± 0.003 ^a^	0.38 ± 0.002 ^ab^	0.35 ± 0.007 ^a^
C22:6 n3	0.006 ± 0.001 ^a^	0.007 ± 0.002 ^a^	0.008 ± 0.004 ^a^	0.003 ± 0.001 ^a^	0.03 ± 0.003 ^b^
C24:0	0.12 ± 0.003 ^ab^	0.13 ± 0.006 ^bc^	0.13 ± 0.002 ^bc^	0.14 ± 0.002 ^c^	0.12 ± 0.001 ^a^

Values are mean ± SE (*n* = 3). Mean values in each row that are associated with different letters are significantly different (one-way ANOVA and Tukey HDS test, *p* < 0.05).

**Table 4:** Fatty acid profile (%) of soy beverages after the in vitro digestion.

Fatty Acid	SB1	SB2	SB3	SB4	SB5
	Bioaccessible Fraction				
C10:0	0.25 ± 0.04 ^b^	0.21 ± 0.02 ^ab^	0.15 ± 0.03 ^ab^	0.25 ± 0.04 ^b^	0.09 ± 0.02 ^a^
C12:0	0.018 ± 0.003 ^a^	0.013 ± 0.001 ^a^	0.015 ± 0.001 ^a^	0.037 ± 0.005 ^b^	0.013 ± 0.001 ^a^
C14:0	0.09 ± 0.009 ^ab^	0.11 ± 0.002 ^b^	0.10 ± 0.012 ^b^	0.11 ± 0.013 ^b^	0.05 ± 0.007 ^a^
C16:0	4.16 ± 0.06 ^a^	6.67 ± 0.53 ^b^	7.43 ± 0.67 ^b^	4.30 ± 0.20 ^a^	3.16 ± 0.49 ^a^
C16:1	0.16 ± 0.004 ^c^	0.14 ± 0.003 ^bc^	0.11 ± 0.004 ^a^	0.22 ± 0.10 ^d^	0.12 ± 0.005 ^ab^
C18:0	2.01 ± 0.06 ^a^	4.06 ± 0.45 ^b^	4.85 ± 0.31 ^b^	2.40 ± 0.19 ^a^	1.63 ± 0.17 ^a^
C18:1 n9	21.3 ± 0.06 ^ab^	23.1 ± 0.19 ^c^	23.1 ± 0.27 ^c^	19.9 ± 0.29 ^a^	21.9 ± 0.62 ^bc^
C18:1 n7	1.15 ± 0.03	1.07 ± 0.04	0.99 ± 0.05	1.08 ± 0.03	1.10 ± 0.06
C18:2 n6	61.8 ± 0.15 ^b^	56.4 ± 0.72 ^a^	54.8 ± 0.76 ^a^	61.9 ± 0.17 ^b^	63.0 ± 1.15 ^b^
C18:3 n6	0.02 ± 0.001	0.02 ± 0.001	0.02 ± 0.001	0.03 ± 0.003	0.03 ± 0.001
C18:3 n3	8.28 ± 0.03 ^cd^	7.09 ± 0.10 ^a^	7.19 ± 0.10 ^ab^	8.89 ± 0.27 ^d^	8.03 ± 0.26 ^bc^
C20:0	0.09 ± 0.004 ^a^	0.21 ± 0.02 ^b^	0.22 ± 0.01 ^b^	0.07 ± 0.005 ^a^	0.11 ± 0.02 ^a^
C20:3 n3	0.008 ± 0.001	0.013 ± 0.001	0.015 ± 0.001	0.010 ± 0.001	0.016 ± 0.003
C20:4 n6	0.03 ± 0.003	0.04 ± 0.002	0.05 ± 0.007	0.05 ± 0.02	0.04 ± 0.004
C20:5 n3	0.03 ± 0.004	0.03 ± 0.001	0.04 ± 0.003	0.04 ± 0.007	0.03 ± 0.009
C22:0	0.12 ± 0.009 ^a^	0.22 ± 0.02 ^b^	0.24 ± 0.009 ^b^	0.08 ± 0.007 ^a^	0.13 ± 0.02 ^a^
C22:6 n3	0.009 ± 0.003	0.02 ± 0.004	0.03 ± 0.009	0.04 ± 0.02	0.02 ± 0.005
C24:0	0.04 ± 0.003 ^a^	0.08 ± 0.006 ^bc^	0.09 ± 0.006 ^c^	0.04 ± 0.005 ^a^	0.06 ± 0.006 ^ab^
	Residual Fraction				
C10:0	0.08 ± 0.02	0.05 ± 0.02	0.05 ± 0.02	0.04 ± 0.007	0.07 ± 0.006
C12:0	0.02 ± 0.001 ^ab^	0.03 ± 0.005 ^b^	0.03 ± 0.001 ^b^	0.01 ± 0.001 ^a^	0.03 ± 0.003 ^b^
C14:0	0.20 ± 0.04 ^ab^	0.37 ± 0.03 ^c^	0.32 ± 0.03 ^bc^	0.12 ± 0.003 ^a^	0.21 ± 0.02 ^ab^
C16:0	13.1 ± 1.02 ^a^	24.9 ± 1.06 ^b^	21.4 ± 1.34 ^b^	10.0 ± 0.48 ^a^	12.2 ± 1.03 ^a^
C16:1	0.15 ± 0.005 ^c^	0.11 ± 0.004 ^ab^	0.10 ± 0.001 ^a^	0.12 ± 0.003 ^b^	0.18 ± 0.003 ^d^
C18:0	5.73 ± 0.44 ^a^	13.9 ± 0.88 ^b^	12.4 ± 1.48 ^b^	3.73 ± 0.25 ^a^	5.60 ± 0.51 ^a^
C18:1 n9	22.4 ± 0.59 ^b^	18.1 ± 0.53 ^a^	19.2 ± 1.16 ^a^	26.4 ± 0.42 ^c^	22.8 ± 0.33 ^b^
C18:1 n7	1.42 ± 0.02	1.09 ± 0.05	1.09 ± 0.09	1.32 ± 0.13	1.42 ± 0.03
C18:2 n6	48.7 ± 0.83 ^b^	34.8 ± 1.37 ^a^	38.3 ± 1.56 ^a^	50.1 ± 0.66 ^b^	48.9 ± 0.98 ^b^
C18:3 n6	0.02 ± 0.001	0.02 ± 0.001	0.02 ± 0.001	0.02 ± 0.001	0.03 ± 0.002
C18:3 n3	6.74 ± 0.15 ^b^	4.35 ± 0.19 ^a^	5.19 ± 0.27 ^a^	6.82 ± 0.11 ^b^	7.09 ± 0.47 ^b^
C20:0	0.30 ± 0.02 ^ab^	0.54 ± 0.02 ^bc^	0.43 ± 0.06 ^c^	0.21 ± 0.02 ^a^	0.23 ± 0.01 ^a^
C20:3 n3	0.01 ± 0.002	0.01 ± 0.005	0.03 ± 0.01	0.01 ± 0.002	0.01 ± 0.001
C20:4 n6	0.06 ± 0.006 ^ab^	0.07 ± 0.004 ^b^	0.07 ± 0.007 ^b^	0.037 ± 0.001 ^a^	0.04 ± 0.005 ^a^
C20:5 n3	0.01 ± 0.00 ^ab^	0.05 ± 0.002 ^b^	0.04 ± 0.001 ^b^	0.01 ± 0.001 ^a^	0.04 ± 0.010 ^b^
C22:0	0.30 ± 0.02 ^ab^	0.47 ± 0.02 ^c^	0.39 ± 0.05 ^bc^	0.22 ± 0.02 ^a^	0.23 ± 0.01 ^a^
C22:6 n3	0.04 ± 0.02	0.13 ± 0.04	0.05 ± 0.03	0.02 ± 0.007	0.13 ± 0.06
C24:0	0.10 ± 0.005 ^ab^	0.19 ± 0.01 ^c^	0.16 ± 0.02 ^bc^	0.08 ± 0.007 ^a^	0.08 ± 0.006 ^a^

Values are mean ± SE (*n* = 3). Mean values in each row that are associated with different letters are significantly different (one-way ANOVA and Tukey test, *p* < 0.05).

**Table 5:** Total phenolic content (TPC) and antioxidant capacity of soy beverages before and after in vitro gastrointestinal digestion (BF).

Beverage	TPC (µmol GAE/mL)		ABTS (µmol TE/mL)		FRAP (µmol TE/mL)	
	Undigested	BF	Undigested	BF	Undigested	BF
SB1	0.91 ± 0.01 ^a^	19.00 ± 0.42 ^b^	1.87 ± 0.04 ^b^	5.22 ± 0.45 ^a^	0.51 ± 0.04 ^ab^	2.91± 0.11 ^bc^
SB2	0.94 ± 0.03 ^ab^	17.70 ± 0.41 ^b^	1.85 ± 0.01 ^b^	5.37 ± 0.21 ^a^	0.39 ± 0.01 ^a^	2.71 ± 0.03 ^b^
SB3	1.10 ± 0.04 ^b^	18.06 ± 0.68 ^b^	2.13 ± 0.07 ^c^	5.37 ± 0.20 ^a^	0.52 ± 0.01 ^ab^	2.86 ± 0.12 ^bc^
SB4	1.29 ± 0.09 ^c^	18.96 ± 0.88 ^b^	2.53 ± 0.01 ^d^	4.97 ± 0.21 ^a^	0.65 ± 0.08 ^b^	3.01 ± 0.08 ^c^
SB5	0.95 ± 0.01 ^ab^	13.77 ± 1.09 ^a^	0.74 ± 0.02 ^a^	5.47 ± 0.32 ^a^	0.37 ± 0.02 ^a^	2.17 ± 0.09 ^a^

Values are mean ± SD (*n* = 3). Mean values in the same column that are associated with different letters indicate significant differences between samples (one-way ANOVA followed by Tukey HDS test, *p* < 0.05). For each analyzed beverage, results after digestion were always significantly higher than before digestion (one-way ANOVA followed by Tukey HDS test, *p* < 0.05).

## Data Availability

The data presented in this study are available on request from the corresponding authors.
